# Biochemical Properties of Synaptic Proteins Are Dependent on Tissue Preparation: NMDA Receptor Solubility Is Regulated by the C‐Terminal Tail

**DOI:** 10.1002/jcb.30664

**Published:** 2024-10-06

**Authors:** Sehoon Won, Colin L. Sweeney, Katherine W. Roche

**Affiliations:** ^1^ Receptor Biology Section National Institute of Neurological Disorders and Stroke, National Institutes of Health Bethesda Maryland USA; ^2^ Genetic Immunotherapy Section National Institute of Allergy and Infectious Diseases, National Institutes of Health Bethesda Maryland USA

**Keywords:** AMPAR, MAGUK, NLGN, NMDAR, PSD, solubility

## Abstract

Synaptic proteins are essential for neuronal development, synaptic transmission, and synaptic plasticity. The postsynaptic density (PSD) is a membrane‐associated structure at excitatory synapses, which is composed of a huge protein complex. To understand the interactions and functions of PSD proteins, researchers have employed a variety of imaging and biochemical approaches including sophisticated mass spectrometry. However, the field is lacking a systematic comparison of different experimental conditions and how they might influence the study of the PSD interactome isolated from various tissue preparations. To evaluate the efficiency of several common solubilization conditions, we isolated receptors, scaffolding proteins, and adhesion molecules from brain tissue or primary cultured neurons or human forebrain neurons differentiated from induced pluripotent stem cells (iPSCs). We observed some striking differences in solubility. We found that N‐methyl‐d‐aspartate receptors (NMDARs) and PSD‐95 are relatively insoluble in brain tissue, cultured neurons, and human forebrain neurons compared to α‐amino‐3‐hydroxy‐5‐methyl‐4‐isoxazolepropionic receptors (AMPARs) or SAP102. In general, synaptic proteins were more soluble in primary neuronal cultures and human forebrain neurons compared to brain tissue. Interestingly, NMDARs are relatively insoluble in HEK293T cells suggesting that insolubility does not directly represent the synaptic fraction but rather it is related to a detergent‐insoluble fraction such as lipid rafts. Surprisingly, truncation of the intracellular carboxyl‐terminal tail (C‐tail) of NMDAR subunits increased NMDAR solubility in HEK293T cells. Our findings show that detergent, pH, and temperature are important for protein preparations to study PSD protein complexes, and NMDAR solubility is regulated by its C‐tail, thus providing a technical guide to study synaptic interactomes and subcellular localization of synaptic proteins.

## 1 Introduction

Neuronal development and synaptic plasticity depend upon the precise localization of synaptic proteins at pre‐ and postsynaptic sites. There are many classes of adhesion molecules, receptors, and scaffolding proteins that are essential for normal brain and synaptic function. Indeed, rare variants identified in many synaptic proteins are pathogenic and underlie neurodevelopmental disorders (NDDs) such as autism spectrum disorder (ASD), intellectual disability (ID), and epilepsy [1, 2, 3, 4, 5, 6]. Therefore, there is an intense focus on understanding how different classes of synaptic proteins function and how they work together for efficient synaptic transmission. The postsynaptic density (PSD) consists of a very large protein–protein network made up of hundreds of proteins [7, 8]. Our understanding of the different classes of proteins expressed at the PSD and how their interactions are dynamically regulated yield insight into synaptic plasticity and synaptic dysfunction.

Historically, a variety of experimental approaches including biochemistry, electrophysiology, and microscopy have been applied to the study of synaptic protein structure, localization, and function [9, 10, 11, 12, 13]. As experimental research tools have become more sophisticated, the standard repertoire of techniques has expanded to include optogenetics, in vivo imaging, mass spectrometry, and RNA sequencing (RNAseq) [14, 15, 16, 17]. The most significant advance in the biochemical characterization of synaptic proteins is the use of sophisticated mass spectrometry to reveal synaptic interactomes [18, 19, 20, 21, 22]. However, as techniques such as mass spectrometry become more prevalent, it is important to note that this approach depends on both the starting preparation (e.g., cell culture vs. brain tissue) and the biochemical parameters used to solubilize the proteins.

Classic studies on the PSD revealed that many postsynaptic proteins are highly insoluble [23, 24, 25, 26]. In fact, the biochemical approaches used to purify the PSD consist of multiple rounds of more stringent washes and thus the PSD is defined by its insolubility [27, 28]. However, approaches such as mass spectrometry used to study postsynaptic protein complexes depend upon solubilizing proteins while maintaining critical protein interactions [20, 21, 29]. To better understand the conditions needed to efficiently solubilize postsynaptic proteins, we characterized two subtypes of glutamate receptors (AMPARs and NMDARs), two members of the PSD‐95 family of scaffolding proteins (PSD‐95 and SAP102), two isoforms of the neuroligin family of adhesion molecules (NLGN1 and NLGN2), a protein tyrosine kinase Fyn and a brain‐specific protein tyrosine phosphatase STEP_61_. We evaluated parameters such as choice of detergent, pH, and temperature and the effects on solubilization of proteins from human forebrain neurons from iPSCs as well as three common tissue preparations: mouse brain lysate, rat primary neuronal cultures, and proteins expressed in HEK293T cells. We identify distinct biochemical properties between the various synaptic proteins and between preparations that are critical factors when designing experiments such as mass spectrometry to study synapses.

## 2 Materials and Methods

### 2.1 cDNA Constructs and Antibodies

Flag‐tagged GluN2A, GluN2B, GluA1, GluA2, YFP‐tagged GluN1, HA‐tagged NLGN1, and NLGN2 cDNA were used to transfect into HEK293T cells. A truncation mutant of GluN2B was deleted by 11 amino acids (Δ11) or 644 amino acids (ΔC) by introducing a stop codon at amino acid of Y_1472_ or E_839_. Antibodies were purchased as follows: Rabbit anti‐GluN2A (cat. M264; Sigma), rabbit anti‐GluN2A (cat. ab124913; Abcam), mouse anti‐GluN2B (cat. Clone N59/36; NeuroMab), rabbit anti‐GluN2B (cat. ab65783; Abcam), mouse anti‐GluN1 [20], rabbit anti‐GluN1 (cat. 109182; Abcam), rabbit anti‐GluA1 [20], mouse anti‐GluA2 (cat. clone L21/32; NeuroMab), mouse anti‐NLGN1 (cat. 129111; Synaptic Systems), rabbit anti‐NLGN2 (cat. 129203; Synaptic Systems), mouse anti‐PSD‐95 (cat. clone K28/43; NeuroMab), mouse anti‐SAP102 (cat. clone N19/2; NeuroMab), mouse anti‐STEP (cat. NB300‐202; Novagen), rabbit anti‐Fyn (cat. 4023; Cell Signaling), mouse anti‐β‐actin (cat. G043; abm), mouse anti‐Flag (cat. F1804; Sigma), rabbit anti‐GFP (cat. A11122; Invitrogen).

### 2.2 Protein Preparation

Proteins were isolated from wild‐type (WT) adult mouse hippocampi tissues, rat cultured cortical neurons, or human forebrain neurons differentiated from iPSCs or HEK293T cells using five different lysis buffers: Triton X‐100 (50 mM Tris‐HCl [pH 7.4], 150 mM NaCl, 1 mM EDTA, 1% Tx‐100), RIPA (50 mM Tris‐HCl [pH 7.5], 150 mM NaCl, 1 mM EDTA, 1% NP‐40, 0.5% sodium deoxycholate [Doc], 0.1% SDS), Doc (50 mM Tris‐HCl [pH 8.0], 150 mM NaCl, 1 mM EDTA, 1% sodium deoxycholate [Doc]), Doc (50 mM Tris‐HCl [pH 8.8], 10 mM EDTA, 1% sodium deoxycholate [Doc]), SDS (50 mM Tris‐HCl [pH 7.5], 5 mM EDTA, 1% SDS). Adult mouse hippocampal tissue was homogenized in lysis buffer using a glass Teflon homogenizer and incubated at 4°C or 37°C for 0.5–1 h after brief sonication, then centrifuged at 100 000×*g* at 4°C for 20 min. The supernatants were saved for immunoblotting. Protein samples were incubated at 42°C for 20 min (Figure S1), instead of boiling at 95°C for 5 min. Cultured cortical neurons, human forebrain neurons, and HEK293T cells were collected using PBS and then proteins were prepared as described for hippocampal tissues.

### 2.3 Animals and Primary Neuronal Culture

The National Institute of Neurological Disorders and Stroke Animal Care and Use Committee approved our use of experimental animals (protocol #1171). C57BL/6 mice and rats were housed in a specific‐pathogen‐free and well‐ventilated facility at the National Institute of Neurological Disorders and Stroke. All animals were handled, and the experiments performed, according to the guidelines of the National Institutes of Health Office of Intramural Animal Care and Use of Laboratory Animals. Mice and rats were housed in a temperature and humidity‐controlled room under a 12‐h light/dark cycle and provided supportive care (i.e. standard diet, drinking water, and diet gel) by animal facility staff. All animals were euthanized in the CO_2_ chamber. Every possible effort and precaution were taken to minimize pain to the animal throughout the experimental procedures.

Adult male mice older than 2 months were euthanized to isolate the hippocampi. Primary rat cortical neuronal cultures were prepared from embryonic Day 18 (E18) embryos of pregnant Sprague–Dawley rats. The cortices were dissected, and the tissue was dissociated for 10 min at 37°C by 0.05% trypsin in 10 mM HEPES‐supplemented HBSS and containing 1.37 mg DNase. Cells were triturated using fire‐polished glass Pasteur pipettes. 9 × 10^6^ cortical neurons were plated on poly‐d‐lysine‐coated 100 mm culture dishes and were cultured in serum‐free Neurobasal medium supplemented with 2% B‐27 and 2 mM l‐glutamine. The cultures were maintained at 37°C in 5% CO_2_/95% air mixture and used at 28 days in vitro (*DIV*) for immunoblotting.

### 2.4 HEK293T Cell Culture and Transfection

HEK293T cells were grown and maintained with 5% FBS and 1% penicillin‐streptomycin in DMEM medium containing high glucose. cDNAs were transfected with polyethyleneimine (PEI) solution (1 mg/mL in ultrapure water). Each plasmid DNA diluted in Opti‐MEM I, to which PEI solution diluted in Opti‐MEM I was added and mixed thoroughly. After a 15 min. incubation at room temperature, HEK293T cells were treated with the mixture and 50 μM APV and 20 mM MgCl_2_ were added 4 h after transfection.

### 2.5 Human Subjects

Blood from healthy volunteers was obtained after written informed consent following the Declaration of Helsinki under the auspices of research protocols approved by the Institutional Review Board of the National Institute of Allergy and Infectious Diseases.

### 2.6 Reprogramming and Culture of Induced Pluripotent Stem Cells (iPSCs)

iPSCs were generated from healthy volunteer peripheral blood CD34^+^ hematopoietic stem/progenitor cells as previously described [30]. Briefly, CD34^+^ cells were purified from cryopreserved peripheral blood mononuclear cells using CD34 microbeads (Miltenyi Biotec, Gaithersburg, MD), then were transduced with non‐integrating CytoTune‐iPS 2.0 Sendai viruses (Thermo Fisher, Waltham, MA) and cultured in Essential 6 medium (Thermo Fisher) supplemented with 100 ng/mL basic fibroblast growth factor (PeproTech, Cranbury, NJ) on plates coated with ESC‐qualified Matrigel (Corning, Corning, NY) for initial formation of iPSC colonies. After establishment, iPSCs were maintained on Matrigel‐coated plates in mTeSR Plus medium (STEMCELL Technologies, Vancouver, BC) supplemented with Primocin antimicrobial reagent (InvivoGen, San Diego, CA).

### 2.7 Differentiation of iPSCs Into Forebrain Neurons

Following the references [31, 32], iPSCs were induced to neural progenitor cells (NPCs) using neural induction medium I for 6 days and then neural induction medium II for 5 days. NPCs were maintained and expanded on Matrigel‐coated cell culture dishes in STEMdiff neural progenitor medium (STEMCELL Technologies). The medium was exchanged every day for fresh NPC medium. For future studies using NPCs, NPCs frozen stocks were made with 20% DMSO/80% FBS in liquid nitrogen tank. NPCs were differentiated into forebrain neurons for 2 months using neural differentiation medium (DMEM/F‐12 supplemented with N2, B‐27 supplement, brain‐derived neurotrophic factor (BDNF) (20 ng/ml, GenScript), glial‐derived neurotrophic factor (GDNF) (20 ng/ml, GenScript), laminin (1 μg/ml, Thermo Fisher). Half the medium was exchanged for fresh medium every 2‐3 days.

### 2.8 Statistical Analysis

All results represent the mean ± SEM from at least three independent experiments. Band densities of immunoblotting were measured using ImageJ software and the statistical significance between samples was calculated using Student's *t*‐test (*n* = number of independent experiments) and was considered significant at *p* < 0.05.

## 3 Results

### 3.1 Synaptic Proteins Vary in Solubility in Hippocampal Tissues

For immunoblotting using transmembrane proteins, previous studies have shown that the temperature can cause protein aggregation [33, 34]. Therefore, we first examined whether the temperature (42°C or 95°C) affects the synaptic protein preparation for immunoblotting in this study (Figure S1). We found that boiling protein samples at 95°C is less effective for detecting NMDARs than incubating at 42°C.

**Figure 1:**
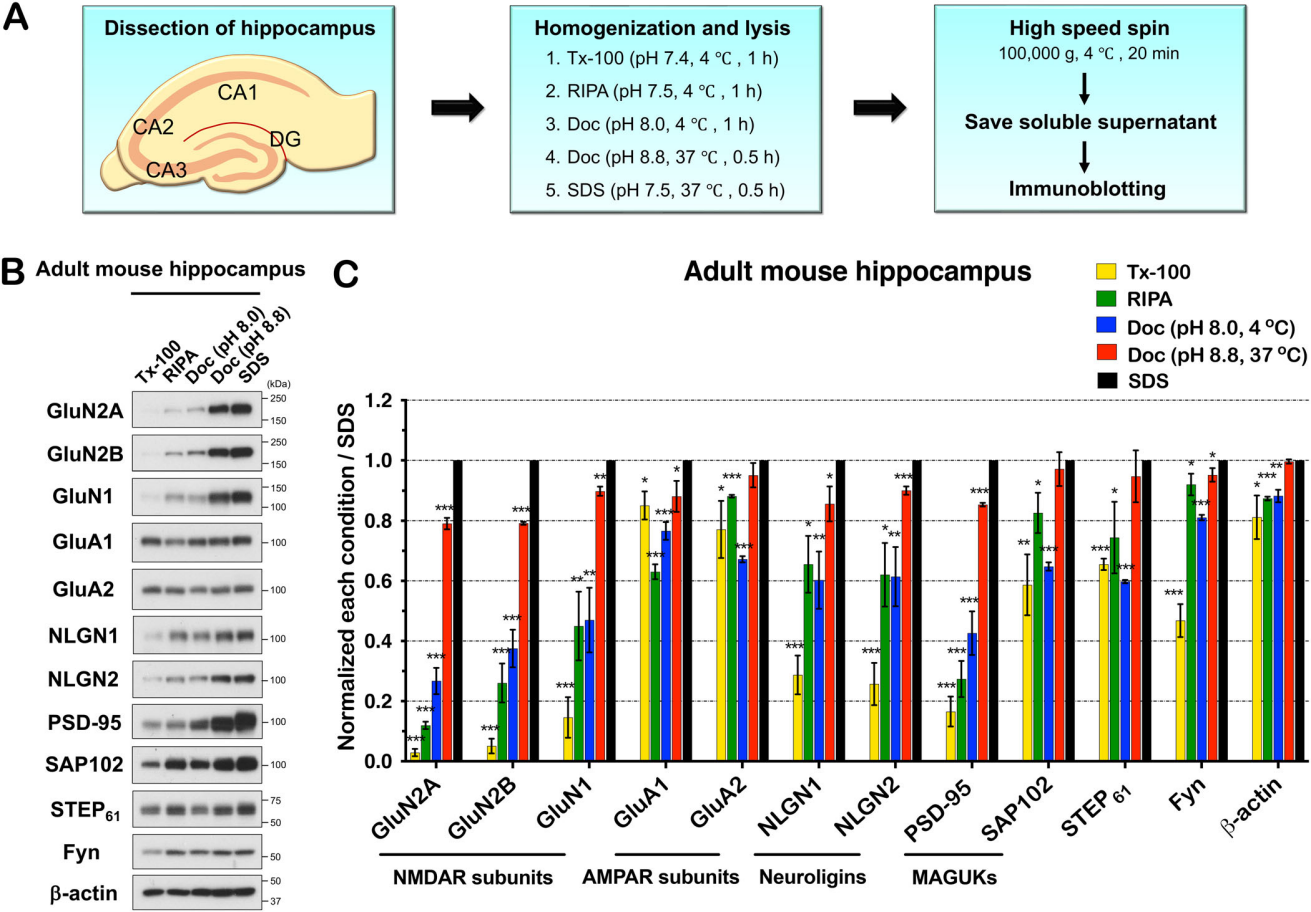
A variety of lysis conditions in mouse brain tissue shows distinct synaptic protein solubility. (A) A schematic synaptic protein isolation. After dissection of hippocampi from WT adult mouse, the tissue was homogenized and lysed in 5 different lysis conditions, centrifuged and soluble supernatant was obtained as described in Materials and methods. (B) Synaptic proteins were immunoblotted with indicated antibodies. (C) Quantification of blots were normalized to SDS (*n* = 3, independent experiments). All blots were normalized to SDS in each protein. Tx‐100 (*p* = 7.2E−08), RIPA (*p* = 1.1E−07), Doc (pH 8.0) (*p* = 3.7E−05), Doc (pH 8.8) (*p* = 0.2E−03) in GluN2A. Tx‐100 (*p* = 1.3E−06), RIPA (*p* = 0.2E−03), Doc (pH 8.0) (*p* = 0.3E−03), Doc (pH 8.8) (*p* = 7.8E−07) in GluN2B. Tx‐100 (*p* = 0.1E−03), RIPA (*p* = 0.004), Doc (pH 8.0) (*p* = 0.004), Doc (pH 8.8) (*p* = 0.001) in GluN1. Tx‐100 (*p* = 0.017), RIPA (*p* = 5.8E−05), Doc (pH 8.0) (*p* = 0.7E−03), Doc (pH 8.8) (*p* = 0.041) in GluA1. Tx‐100 (*p* = 0.037), RIPA (*p* = 5.5E−06), Doc (pH 8.0) (*p* = 2.9E−06), Doc (pH 8.8) (*p* = 0.145) in GluA2. Tx‐100 (*p* = 0.2E−03), RIPA (*p* = 0.011), Doc (pH 8.0) (*p* = 0.007), Doc (pH 8.8) (*p* = 0.034) in NLGN1. Tx‐100 (*p* = 0.2E−03), RIPA (*p* = 0.011), Doc (pH 8.0) (*p* = 0.009), Doc (pH 8.8) (*p* = 0.9E−03) in NLGN2. Tx‐100 (*p* = 3.7E−05), RIPA (*p* = 0.1E−03), Doc (pH 8.0) (*p* = 0.7E−03), Doc (pH 8.8) (*p* = 8.2E−06) in PSD‐95. Tx‐100 (*p* = 0.008), RIPA (*p* = 0.03), Doc (pH 8.0) (*p* = 6.9E−06), Doc (pH 8.8) (*p* = 0.317) in SAP102. Tx‐100 (*p* = 2.6E−05), RIPA (*p* = 0.049), Doc (pH 8.0) (*p* = 9.6E−08), Doc (pH 8.8) (*p* = 0.287) in STEP_61_. Tx‐100 (*p* = 0.3E−03), RIPA (*p* = 0.046), Doc (pH 8.0) (*p* = 1.6E−05), Doc (pH 8.8) (*p* = 0.049) in Fyn. Tx‐100 (*p* = 0.03), RIPA (*p* = 1.9E−05), Doc (pH 8.0) (*p* = 0.002), Doc (pH 8.8) (*p* = 0.308) in β‐actin. Error bars represent ± SEM, * *p* < 0.05, ** *p* < 0.01, *** *p* < 0.001.

To investigate synaptic protein solubility in brain tissue, we dissected hippocampi from WT adult mouse brain and isolated proteins under different conditions. We compared the effects of pH (7.4 or 8.8), temperature (4°C or 37°C) in five different lysis buffers (please see the details in Materials and Methods). The five different conditions are shown in Figure 1A and the following indicate the kind of lysis buffer, lysis temperature, and incubation time: 1. Triton X‐100 (Tx‐100) lysis buffer at 4°C for 1 h; 2. Radioimmunoprecipitation assay (RIPA) lysis buffer at 4°C for 1 h; 3. Sodium deoxycholate (Doc) lysis buffer (pH 8.0) at 4°C for 1 h; 4. High pH Doc lysis buffer (pH 8.8) at 37°C for 0.5 h; and 5. Sodium dodecyl sulfate (SDS) lysis buffer at 37°C for 0.5 h. We performed immunoblotting using antibodies against various endogenous synaptic proteins including N‐methyl‐d‐aspartate receptor (NMDAR) subunits, GluN2A, GluN2B, and GluN1; α‐amino‐3‐hydroxy‐5‐methyl‐4‐isoxazolepropionic acid receptor (AMPAR) subunits, GluA1 and GluA2; postsynaptic cell adhesion proteins, neuroligin‐1 (NLGN1) and NLGN2; membrane‐associated guanylate kinase (MAGUK) proteins, postsynaptic density protein 95 (PSD‐95) and synapse‐associated protein 102 (SAP102); a tyrosine protein phosphatase, Striatal‐enriched protein tyrosine phosphatase 61 (STEP_61_); a member of the protein tyrosine kinase oncogene family, Fyn; and β‐actin (Figure 1B). We observed striking differences in the solubility between different synaptic proteins, with NMDAR subunits and MAGUK family members requiring the most stringent condition for efficient solubilization.

**Figure 2:**
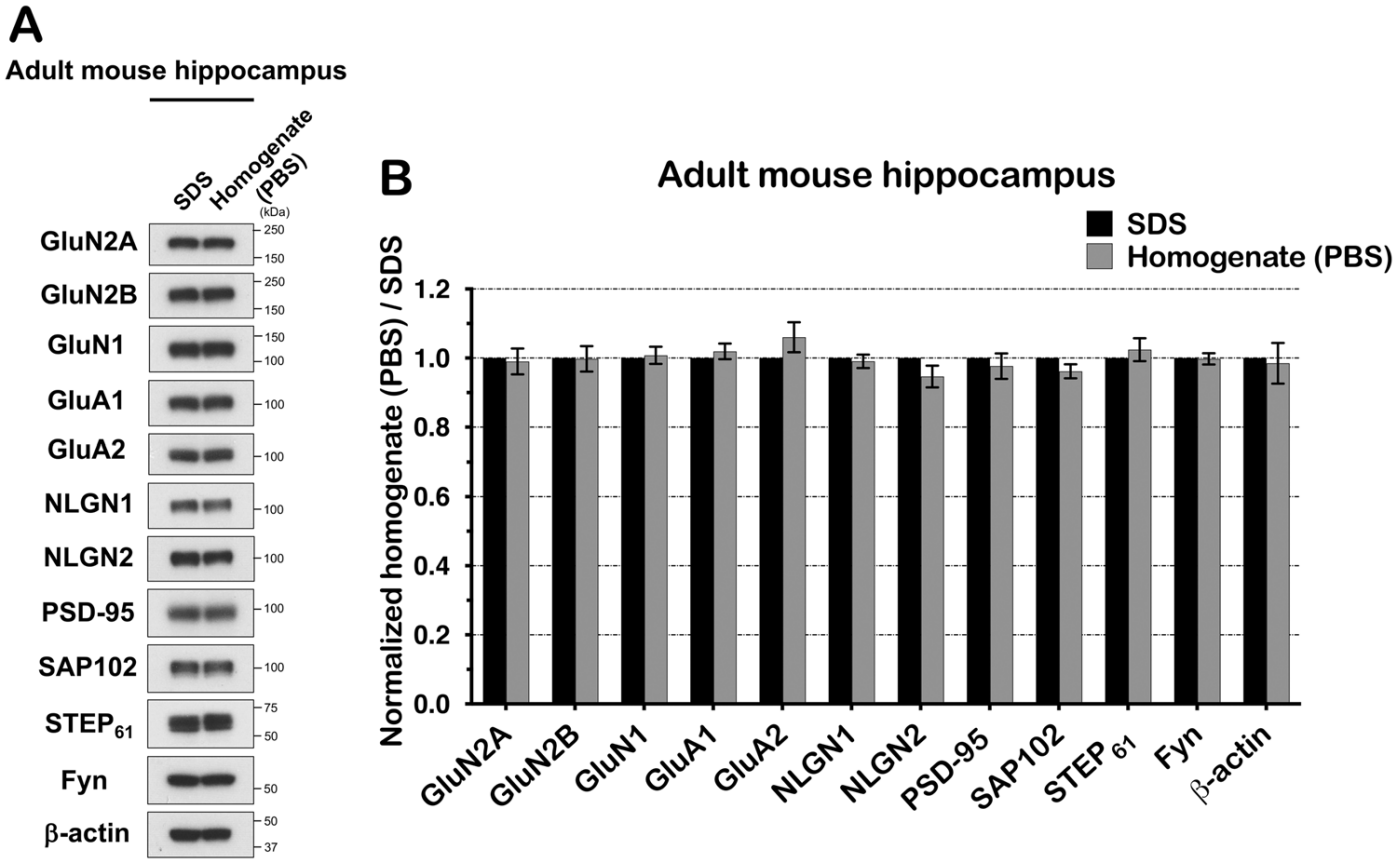
SDS lysis buffer isolates total protein from adult mouse brain hippocampal tissues. (A) Hippocampi in WT adult mouse brain were dissected, homogenized, and lysed in SDS lysis buffer. The hippocampi were homogenized in PBS. The proteins from homogenate were resuspended in PBS and the lysate in SDS were immunoblotted. Experiment was repeated three times independently. (B) The band intensity of each protein was quantified and analyzed, then compared to the SDS condition. GluN2A (*p* = 0.404), GluN2B (*p* = 0.476), GluN1 (*p* = 0.382), GluA1 (*p* = 0.221), GluA2 (*p* = 0.119), NLGN1 (*p* = 0.325), NLGN2 (*p* = 0.081), PSD‐95 (*p* = 0.282), SAP102 (*p* = 0.064), STEP_61_ (*p* = 0.252), Fyn (*p* = 0.444), and β‐actin (*p* = 0.404) in homogenate. All proteins immunoblotted in both homogenate in PBS and the lysate in SDS condition did not show a statistically significant difference.

The most profound differences were found with Triton X‐100 solubilization, which is commonly used as a detergent to solubilize membrane proteins while leaving the protein interactions intact. We found that the NMDAR subunits GluN2A, GluN2B, and GluN1 were highly insoluble in Triton X‐100 lysis buffer compared to AMPAR subunits. This difference is consistent with earlier studies [29, 35] and stood out as the most distinct biochemical difference in this detergent. The levels of GluN2A and GluN2B solubilized in Triton X‐100 lysis buffer were less than 6% relative to SDS lysis buffer, which we used as a control for total solubilization (Figure 2). Interestingly, in two different Doc lysis conditions, solubility of NMDAR subunits was markedly different. Indeed, we observed that solubilization of NMDAR subunits was much more efficient in the condition combining high pH and higher temperature (Doc pH 8.8 at 37°C) relative to Doc pH 8.0, 4°C (GluN2A: 26.7% vs. 79.1%; GluN2B: 37.5% vs. 79.2%; and GluN1: 47% vs. 89.8% in Doc pH 8.0 at 4°C vs. pH 8.8 at 37°C, respectively) (Figure 1C). Thus, pH and temperature are especially important parameters for NMDAR solubility compared to other synaptic proteins. In contrast, the AMPAR subunits, GluA1 and GluA2, were much more soluble than other synaptic proteins, even in Triton X‐100 lysis conditions (85.1% and 77.1%, respectively) (Table 1). Notably, GluA1 was more soluble in Triton X‐100 than in RIPA buffer, which was the only such example.

**Table 1:** Percentage ratio of synaptic proteins in hippocampal tissue using five lysis conditions shown in Figure 1C.

Lysis condition	GluN2A	GluN2B	GluN1	GluA1	GluA2	NLGN1	NLGN2	PSD‐95	SAP102	STEP_61_	Fyn	β‐actin
Tx‐100	2.9 ± 1.2	5.1 ± 2.4	14.6 ± 6.8	85.1 ± 4.7	77.1 ± 9.5	28.7 ± 6.5	25.7 ± 7.0	16.5 ± 5.0	58.7 ± 10.1	65.5 ± 1.9	46.8 ± 5.5	81.2 ± 7.2
RIPA	11.9 ± 1.2	26 ± 6.5	45 ± 11.4	63 ± 2.5	88.2 ± 0.4	65.5 ± 9.5	62.1 ± 10.6	27.4 ± 6.0	82.6 ± 6.7	74.4 ± 11.9	92.1 ± 3.6	87.4 ± 0.6
Doc (pH 8.0, 4°C)	26.7 ± 4.4	37.5 ± 6.3	47 ± 10.8	76.6 ± 2.9	67.2 ± 1.0	60.3 ± 9.5	61.4 ± 9.9	42.6 ± 7.2	64.8 ± 1.4	59.8 ± 0.5	81.1 ± 0.9	88.3 ± 2.1
Doc (pH 8.8, 37°C)	79.1 ± 1.9	79.2 ± 0.5	89.8 ± 1.5	88.1 ± 5.2	95.1 ± 4.0	85.6 ± 5.8	90.1 ± 1.4	85.4 ± 0.6	97.1 ± 5.6	94.8 ± 8.6	95.2 ± 2.2	99.6 ± 0.7
SDS	100	100	100	100	100	100	100	100	100	100	100	100

*Note:* Each value indicates an average ± SEM.

Like NMDARs, the PSD‐95 family of proteins had differing solubility among family members. PSD‐95 solubility in Tx‐100 lysis buffer was 16.5% of the protein level in SDS lysis buffer. In contrast, SAP102 showed higher solubility (58.7%) in Tx‐100 lysis buffer (Figure 1C and Table 1) compared to PSD‐95, consistent with earlier studies [23, 29, 36, 37]. Notably, the difference in RIPA solubility between PSD‐95 and SAP102 was even more pronounced than with Triton X‐100. Similar to NMDAR subunits, the solubility of PSD‐95 under various lysis conditions revealed dramatic differences depending on detergent, pH, and temperature. PSD‐95 is a palmitoylated protein and is localized at the center of excitatory synapses [38, 39, 40], whereas SAP102 is not palmitoylated and predominantly localized extrasynaptically [41] and this is consistent with PSD‐95 being more insoluble.

The neuroligins (NLGN1 and NLGN2) showed 28.7% and 25.7% solubility in Tx‐100 lysis buffer, whereas they were much more soluble in RIPA and Doc (Figure 1C). STEP_61_ and Fyn showed moderate solubility. The protein solubility of STEP_61_ was 65.5% in Tx‐100, 74.4% in RIPA, and 59.8% in Doc lysis buffers at pH 8.0, whereas it showed almost complete solubility (94.8%) in Doc at pH 8.8 (Table 1). The membrane‐anchored myristoylated protein tyrosine kinase Fyn displayed 46.8% solubility in Tx‐100, but much higher solubility in RIPA and the two Doc lysis buffers. In contrast to the synaptic proteins analyzed, β‐actin was mostly soluble in all detergents: Tx‐100, RIPA, and Doc (pH 8.0, 4°C) lysis buffers isolated 81.2%, 87.4%, and 88.3% protein, respectively.

### 3.2 1% SDS Lysis Buffer Condition in Mouse Hippocampal Tissue Isolates Total Proteins

We used five different lysis conditions to isolate proteins from mouse hippocampal tissue. The strongest lysis condition is using 1% SDS at 37°C. To validate the SDS lysis buffer as the most stringent condition, we isolated total protein homogenate from mouse hippocampus in PBS. Then we directly compared the total homogenates to the SDS‐solubilized lysates. We performed immunoblotting for GluN2A, GluN2B, GluN1, GluA1, GluA2, NLGN1, NLGN2, PSD‐95, SAP102, STEP_61_, and Fyn compared to β‐actin. In all cases, the SDS lysis condition was not statistically different from the total homogenate (Figure 2). Therefore, we conclude that the SDS lysis buffer condition completely solubilizes the proteins from mouse hippocampal tissue.

### 3.3 Temperature and pH Influence on NMDAR Protein Solubility in Mouse Hippocampal Tissue

**Figure 3:**
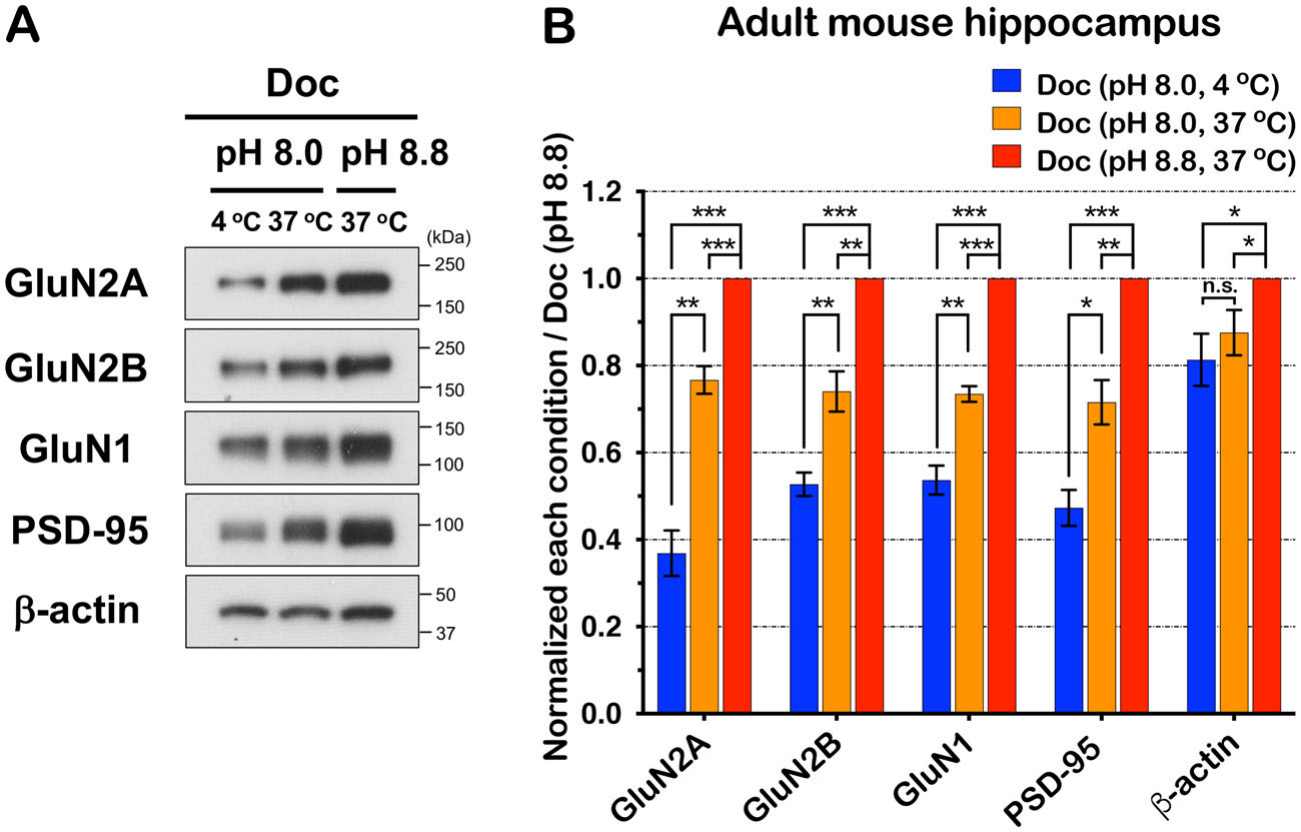
Temperature and pH affect solubility of NMDAR and PSD‐95. (A) NMDAR subunits and PSD‐95 in different temperature and pH lysis conditions containing Doc were isolated and immunoblotted. (B) Analysis of each band intensity and normalization with the pH 8.8 Doc lysis buffer at 37°C condition (*n* = 3, independent experiments). All blots were normalized to Doc (pH 8.8, 37°C). Doc (pH 8.0, 4°C) (*p* = 0.4E−03), Doc (pH 8.0, 37°C) (*p* = 0.3E−03) in GluN2A. Doc (pH 8.0, 4°C) (*p* = 3.1E−05), Doc (pH 8.0, 37°C) (*p* = 0.002) in GluN2B. Doc (pH 8.0, 4°C) (*p* = 7.8E−05), Doc (pH 8.0, 37°C) (*p* = 6.0E−05) in GluN1. Doc (pH 8.0, 4°C) (*p* = 0.1E−03), Doc (pH 8.0, 37°C) (*p* = 0.003) in PSD‐95. Doc (pH 8.0, 4°C) (*p* = 0.018), Doc (pH 8.0, 37°C) (*p* = 0.038) in β‐actin. Doc (pH 8.0, 4°C) was compared to Doc (pH 8.0, 37°C). *p* = 0.003 in GluN2A, *p* = 0.008 in GluN2B, *p* = 0.003 in GluN1, *p* = 0.010 in PSD‐95, *p* = 0.237 in β‐actin. Error bars represent ± SEM, * *p* < 0.05, ** *p* < 0.01, *** *p* < 0.001, n.s.: not significant, *p* value is compared to the Doc condition (pH 8.8, 37°C).

Because the solubility of NMDAR subunits and PSD‐95 vary significantly based on detergent, temperature, and pH, we compared additional conditions. We isolated proteins in Doc at different temperatures and pH, since this detergent seemed to be more efficient in solubilizing these proteins relative to Tx‐100 and RIPA buffer. For both NMDARs and PSD‐95 we observed a significant increase in solubilization in conditions that combined higher pH and higher temperature (Figure 3). In contrast, temperature and pH appeared to have little effect on actin solubility.

### 3.4 The Solubility of Synaptic Proteins Is Increased in Cultured Neurons

**Figure 4:**
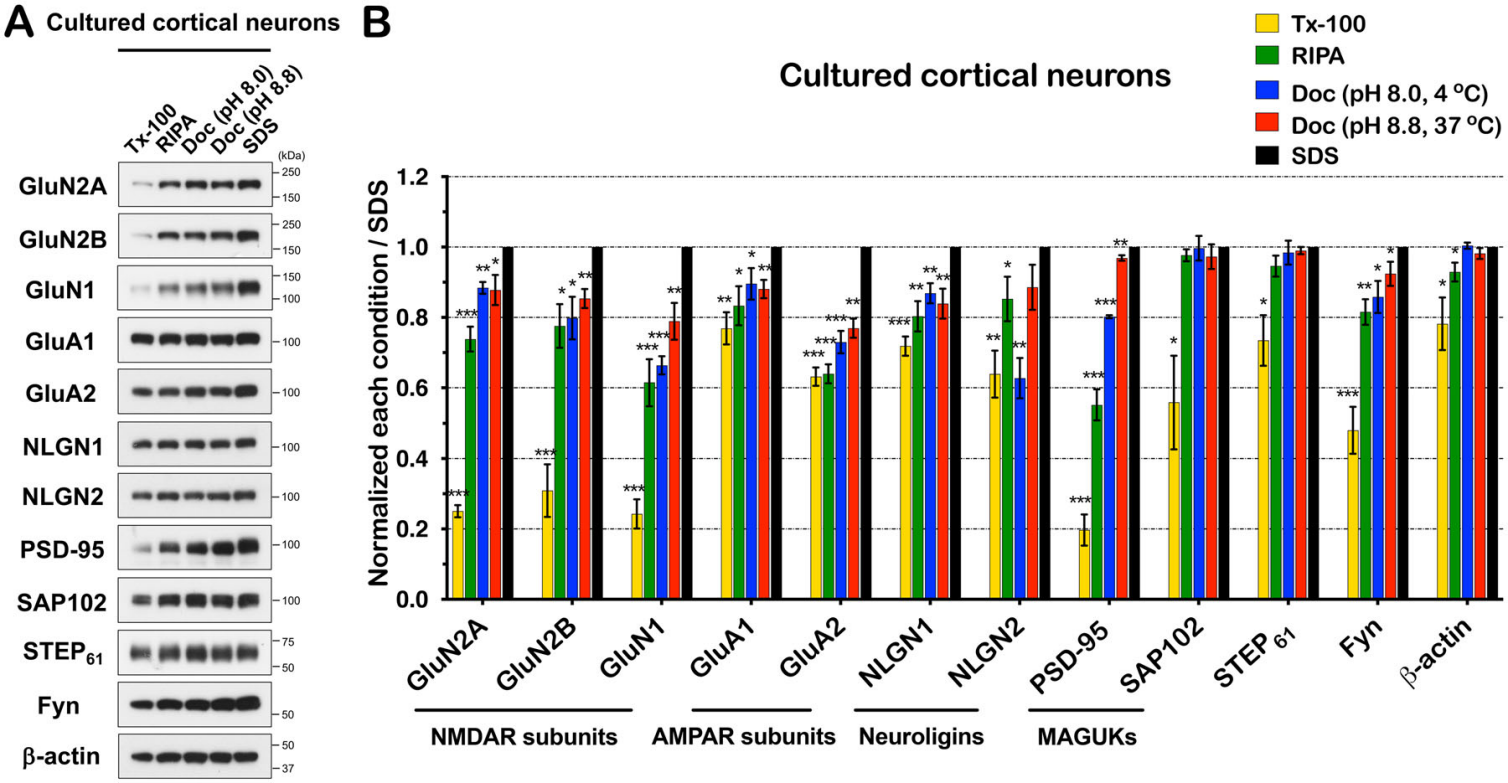
NMDAR subunits from cultured cortical neurons are more soluble compared to hippocampal tissue. (A) Synaptic proteins were isolated from cultured cortical neurons at *DIV* 28. Equal amounts of protein were immunoblotted with the indicated antibodies and the result was quantified, and analyzed statistically (*n* = 3, independent experiments). (B) All blots were normalized to the SDS condition for each protein. Tx‐100 (*p* = 8.4E−07), RIPA (*p* = 0.8E−03), Doc (pH 8.0) (*p* = 0.001), Doc (pH 8.8) (*p* = 0.023) in GluN2A. Tx‐100 (*p* = 0.4E−03), RIPA (*p* = 0.011), Doc (pH 8.0) (*p* = 0.014), Doc (pH 8.8) (*p* = 0.003) in GluN2B. Tx‐100 (*p* = 2.7E−05), RIPA (*p* = 0.002), Doc (pH 8.0) (*p* = 9.9E−05), Doc (pH 8.8) (*p* = 0.008) in GluN1. Tx‐100 (*p* = 0.004), RIPA (*p* = 0.02), Doc (pH 8.0) (*p* = 0.04), Doc (pH 8.8) (*p* = 0.005) in GluA1. Tx‐100 (*p* = 7.2E−05), RIPA (*p* = 9.1E−05), Doc (pH 8.0) (*p* = 0.5E−03), Doc (pH 8.8) (*p* = 0.5E−03) in GluA2. Tx‐100 (*p* = 0.3E−03), RIPA (*p* = 0.005), Doc (pH 8.0) (*p* = 0.004), Doc (pH 8.8) (*p* = 0.009) in NLGN1. Tx‐100 (*p* = 0.003), RIPA (*p* = 0.04), Doc (pH 8.0) (*p* = 0.001), Doc (pH 8.8) (*p* = 0.07) in NLGN2. Tx‐100 (*p* = 2.7E−05), RIPA (*p* = 0.2E−03), Doc (pH 8.0) (*p* = 5.9E−07), Doc (pH 8.8) (*p* = 0.008) in PSD‐95. Tx‐100 (*p* = 0.015), RIPA (*p* = 0.121), Doc (pH 8.0) (*p* = 0.467), Doc (pH 8.8) (*p* = 0.241) in SAP102. Tx‐100 (*p* = 0.01), RIPA (*p* = 0.071), Doc (pH 8.0) (*p* = 0.334), Doc (pH 8.8) (*p* = 0.192) in STEP_61_. Tx‐100 (*p* = 0.7E−03), RIPA (*p* = 0.004), Doc (pH 8.0) (*p* = 0.018), Doc (pH 8.8) (*p* = 0.045) in Fyn. Tx‐100 (*p* = 0.022), RIPA (*p* = 0.029), Doc (pH 8.0) (*p* = 0.333), Doc (pH 8.8) (*p* = 0.152) in β‐actin. Error bars represent ± SEM, *p < 0.05, **p < 0.01, ***p < 0.001, *p* value is compared to the SDS condition.

Primary neuronal cultures are a popular preparation to study synapses and endogenous synaptic proteins. Therefore, we next wanted to evaluate the solubility of synaptic proteins in cultured neurons to compare to brain tissue (Figure 4). To this end, we prepared dissociated cultured cortical neurons, isolated lysates at *DIV* 28, and performed immunoblotting to detect the same proteins as in Figure 1 (Figure 4A). The solubility of GluN2A, GluN2B, GluN1, and PSD‐95 in Tx‐100 lysis buffer was modest (25.1%, 30.9%, 24.3%, and 19.7%) compared to solubilization in SDS lysis buffer (Figure 4B). However, the solubility of these proteins was markedly increased (to 73.8%, 77.6%, 61.5%, and 55.2%) in RIPA lysis buffer. In contrast, GluA1, GluA2, NLGN1, and NLGN2 were moderately soluble in Tx‐100 lysis buffer (76.9%, 63.2%, 71.9%, and 63.9%) and solubility did not dramatically increase in RIPA, with the exception of NLGN2. SAP102 and STEP_61_ were moderately soluble (55.9% and 73.5%) in Tx‐100 lysis buffer, but both had increased solubility in RIPA, matching SDS levels (Table 2). Fyn also showed moderate Triton X‐100 solubility compared to the more stringent conditions. Overall, the solubility of synaptic proteins in cultured cortical neurons is increased compared to hippocampal tissue; however, the choice of lysis conditions can still impact the extent of protein solubilization from this preparation.

**Table 2:** Percentage ratio of synaptic proteins in cultured neurons using five lysis conditions shown in Figure 4B.

Lysis condition	GluN2A	GluN2B	GluN1	GluA1	GluA2	NLGN1	NLGN2	PSD‐95	SAP102	STEP_61_	Fyn	β‐actin
Tx‐100	25.1 ± 1.7	30.9 ± 7.5	24.3 ± 4.2	76.9 ± 4.6	63.2 ± 2.6	71.9 ± 2.7	63.9 ± 6.7	19.7 ± 4.4	55.9 ± 13.3	73.5 ± 7.2	48 ± 6.7	78.2 ± 7.5
RIPA	73.8 ± 3.5	77.6 ± 6.2	61.5 ± 6.6	83.3 ± 5.5	64 ± 2.7	80.3 ± 4.3	85.3 ± 6.3	55.2 ± 4.4	97.7 ± 1.7	94.6 ± 3.0	81.6 ± 3.6	92.9 ± 2.7
Doc (pH 8.0, 4°C)	88.4 ± 1.7	79.8 ± 6.0	66.4 ± 2.6	89.6 ± 4.5	73 ± 3.2	86.9 ± 2.8	62.7 ± 5.7	80.2 ± 0.4	99.7 ± 3.5	98.4 ± 3.4	85.8 ± 4.6	100 ± 0.9
Doc (pH 8.8, 37°C)	87.8 ± 4.3	85.4 ± 2.7	78.9 ± 5.2	88.1 ± 2.6	77 ± 2.7	83.9 ± 4.2	88.6 ± 6.4	96.9 ± 0.8	97.3 ± 3.5	99 ± 1.1	92.4 ± 3.4	98.2 ± 1.6
SDS	100	100	100	100	100	100	100	100	100	100	100	100

*Note:* Each value indicates an average ± SEM.

### 3.5 The Solubility of NMDARs Is More Dependent on Protein Isolation Methods Than the Other Synaptic Proteins

**Figure 5:**
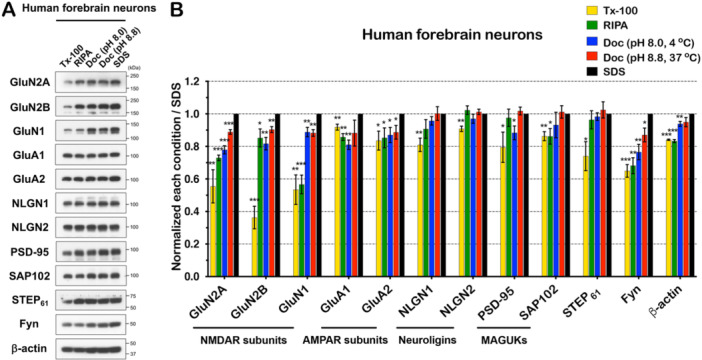
Solubility of NMDAR subunits in human forebrain neurons from iPSCs is dependent on protein preparation methods, whereas PSD‐95 is more soluble in all conditions. (A) Synaptic proteins were isolated from human forebrain neurons at 2‐month differentiation. Equal amounts of protein were immunoblotted with indicated antibodies. (B) The result was quantified and analyzed statistically (*n* = 3, independent experiments). All blots were normalized to the SDS condition for each protein. Tx‐100 (*p* = 0.006), RIPA (*p* = 0.5E−06), Doc (pH 8.0) (*p* = 0.4E−03), Doc (pH 8.8) (*p* = 0.7E−03) in GluN2A. Tx‐100 (*p* = 0.4E−03), RIPA (*p* = 0.028), Doc (pH 8.0) (*p* = 0.004), Doc (pH 8.8) (*p* = 0.003) in GluN2B. Tx‐100 (*p* = 0.003), RIPA (*p* = 0.08E−03), Doc (pH 8.0) (*p* = 0.009), Doc (pH 8.8) (*p* = 0.002) in GluN1. Tx‐100 (*p* = 0.006), RIPA (*p* = 0.001), Doc (pH 8.0) (*p* = 0.001), Doc (pH 8.8) (*p* = 0.106) in GluA1. Tx‐100 (*p* = 0.024), RIPA (*p* = 0.037), Doc (pH 8.0) (*p* = 0.025), Doc (pH 8.8) (*p* = 0.028) in GluA2. Tx‐100 (*p* = 0.005), RIPA (*p* = 0.09), Doc (pH 8.0) (*p* = 0.088), Doc (pH 8.8) (*p* = 0.48) in NLGN1. Tx‐100 (*p* = 0.002), RIPA (*p* = 0.215), Doc (pH 8.0) (*p* = 0.171), Doc (pH 8.8) (*p* = 0.242) in NLGN2. Tx‐100 (*p* = 0.046), RIPA (*p* = 0.338), Doc (pH 8.0) (*p* = 0.025), Doc (pH 8.8) (*p* = 0.249) in PSD‐95. Tx‐100 (*p* = 0.004), RIPA (*p* = 0.022), Doc (pH 8.0) (*p* = 0.216), Doc (pH 8.8) (*p* = 0.387) in SAP102. Tx‐100 (*p* = 0.022), RIPA (*p* = 0.274), Doc (pH 8.0) (*p* = 0.26), Doc (pH 8.8) (*p* = 0.323) in STEP_61_. Tx‐100 (*p* = 0.4E−03), RIPA (*p* = 0.001), Doc (pH 8.0) (*p* = 0.004), Doc (pH 8.8) (*p* = 0.019) in Fyn. Tx‐100 (*p* = 1.3E−06), RIPA (*p* = 3.3E−05), Doc (pH 8.0) (*p* = 0.008), Doc (pH 8.8) (*p* = 0.075) in β‐actin. Error bars represent ± SEM, *p < 0.05, **p < 0.01, ***p < 0.001, *p* value is compared to the SDS condition.

To study human NDDs, a variety of approaches using human neurons including human iPSCs are performed with different induction methods. Among them, we used a multi‐step protocol to induce human forebrain neurons from iPSCs [31, 32] because the rapid single‐step method using NGN‐2 or ASCL‐1 induction is not sufficient for establishment of sophisticated complexes of neuronal synaptic proteins [42, 43]. To investigate the solubility of synaptic proteins, we cultured human forebrain neurons from iPSCs, isolated protein lysates at 2‐month differentiation, and performed immunoblotting to detect the same synaptic proteins as in Figures 1 and 4 (Figure 5). NMDAR subunits (GluN2A: 55.5%, GluN2B: 36.3%, and GluN1: 53.4%) are less soluble than AMPAR subunits (GluA1: 91.9% and GluA2:83.6%), NLGNs (NLGN1: 81% and NLGN2: 90.8%), MAGUKs (PSD‐95: 79.5% and SAP102: 83.6%) in Tx‐100 lysis buffer compared to the solubilization in 1% SDS lysis buffer. The solubility of AMPAR subunits, NLGNs, and MAGUKs in Tx‐100 lysis buffer is up to or higher than 80% of that in SDS lysis buffer. The NMDAR subunit GluN1 (56.6%) and Fyn (68.2%) are still insoluble in RIPA lysis buffer, whereas the solubility of the other proteins showed over 70% (Table 3). Interestingly, synaptic proteins in human forebrain neurons are more soluble in mild lysis buffer such as 1% Tx‐100 lysis buffer and RIPA buffer than in brain tissue (Figure 1 and Table 1). The solubility of synaptic proteins in human forebrain neurons is similar to that in cultured cortical neurons.

**Table 3:** Percentage ratio of synaptic proteins in human forebrain neurons using five lysis conditions shown in Figure 5B.

Lysis condition	GluN2A	GluN2B	GluN1	GluA1	GluA2	NLGN1	NLGN2	PSD‐95	SAP102	STEP_61_	Fyn	β‐actin
Tx‐100	55.5 ± 10.2	36.3 ± 6.9	53.4 ± 9.1	91.9 ± 1.8	83.6 ± 5.8	81.0 ± 4.1	90.8 ± 1.6	79.5 ± 9.3	86.3 ± 2.8	74.0 ± 8.9	65.0 ± 3.9	84.1 ± 0.4
RIPA	73.0 ± 1.7	85.2 ± 5.5	56.6 ± 5.7	85.7 ± 2.1	85.3 ± 6.1	90.7 ± 5.7	102.3 ± 2.6	97.6 ± 5.3	86.2 ± 4.8	96.4 ± 5.6	68.2 ± 4.9	83.3 ± 1.0
Doc (pH 8.0, 4°C)	77.9 ± 2.5	81.7 ± 3.8	88.8 ± 2.9	81.1 ± 2.9	87.0 ± 4.7	95.7 ± 2.6	97.0 ± 2.8	88.4 ± 4.2	93.2 ± 7.8	98.3 ± 2.3	76.5 ± 4.7	93.9 ± 1.5
Doc (pH 8.8, 37°C)	88.9 ± 1.4	90.4 ± 1.8	88.3 ± 2.0	88.2 ± 8.0	88.7 ± 4.2	100.2 ± 4.2	101.2 ± 1.6	101.8 ± 2.4	101.2 ± 3.8	102.5 ± 5.0	87.0 ± 4.2	94.9 ± 2.9
SDS	100	100	100	100	100	100	100	100	100	100	100	100

*Note:* Each value indicates an average ± SEM.

### 3.6 The Solubility of Glutamate Receptors Expressed in Heterologous Cells Differs From Neuronal Tissues or Primary Cultures

**Figure 6:**
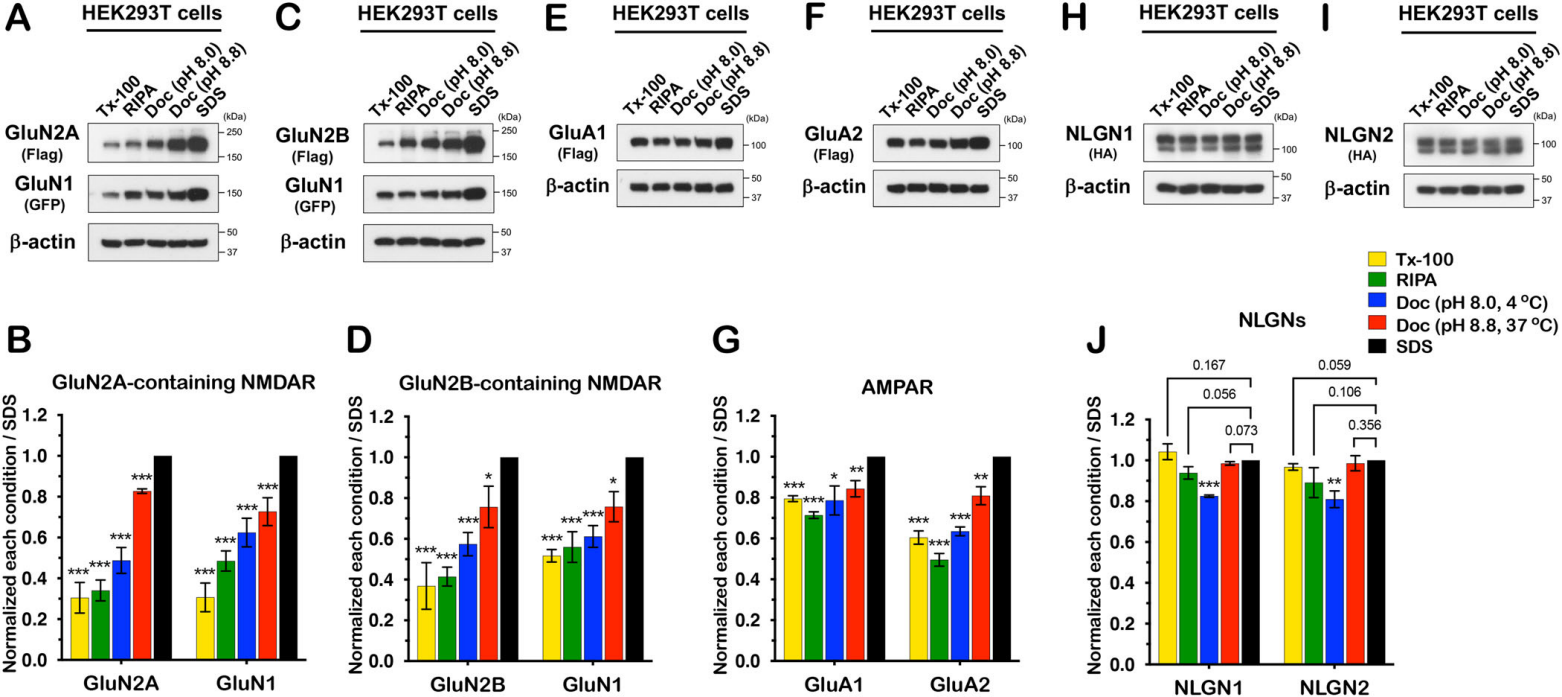
NMDAR solubility is highly dependent on lysis condition compared to AMPARs in heterologous cells. (A–D) GluN1 was cotransfected with GluN2A or 2B into HEK293T cells. After 1 day, proteins were isolated in five different lysis conditions, then immunoblotting for NMDAR subunits was performed using flag or GFP antibody. Each condition was normalized to the SDS condition. In (B), Tx‐100 (*p* = 0.4E−03), RIPA (*p* = 0.1E−03), Doc (pH 8.0) (*p* = 0.6E−03), Doc (pH 8.8) (*p* = 4.7E−05) in GluN2A. Tx‐100 (*p* = 0.3E−03), RIPA (*p* = 0.2E−03), Doc (pH 8.0) (*p* = 0.003), Doc (pH 8.8) (*p* = 0.008) in GluN1. In (D), Tx‐100 (*p* = 0.003), RIPA (*p* = 0.1E−03), Doc (pH 8.0) (*p* = 0.9E−03), Doc (pH 8.8) (*p* = 0.037) in GluN2B. Tx‐100 (*p* = 4.7E−05), RIPA (*p* = 0.002), Doc (pH 8.0) (*p* = 0.9E−03), Doc (pH 8.8) (*p* = 0.015) in GluN1. (E–G) GluA1 or 2 was transfected into HEK293T cells and protein samples were prepared 1 day after transfection, then immunoblotted with indicated antibodies. Each condition was normalized to SDS. In (G), Tx‐100 (*p* = 6.6E−05), RIPA (*p* = 3.3E−05), Doc (pH 8.0) (*p* = 0.019), Doc (pH 8.8) (*p* = 0.008) in GluA1. Tx‐100 (*p* = 0.1E−03), RIPA (*p* = 4.3E−05), Doc (pH 8.0) (*p* = 3.5E−05), Doc (pH 8.8) (*p* = 0.006) in GluA2. All experiments were repeated three times independently and the band intensities were quantified using ImageJ and analyzed statistically. All blots were normalized to the SDS condition. Error bars represent ± SEM, *p < 0.05, **p < 0.01, ***p < 0.001, *p* value is compared to the SDS condition.

Because glutamate receptors are often expressed in heterologous cells to study their properties more directly, we next examined the solubility of receptors in HEK293T cells. We transfected NMDARs or AMPARs in HEK293T cells and isolated proteins using the same conditions (described in Figure 1A). For NMDA receptors, we cotransfected GluN1 with GluN2A or GluN2B, whereas we performed single transfections to study GluA1, GluA2, NLGN1, and NLGN2. We immunoblotted for GluN2A, GluN2B, GluN1 (Figure 6A–D), GluA1, GluA2 (Figure 6E–G), NLGN1, and NLGN2 (Figure 6H–J). Interestingly, NMDAR solubility in HEK293T cells was dramatically different depending on the lysis conditions. For all three NMDAR subunits, the proteins were least soluble in Triton X‐100 and best solubilized in Doc at higher temperature and pH, compared to SDS conditions. In contrast, GluA1 displayed similar solubility in all lysis conditions compared to SDS. GluA2 showed distinct solubility between conditions and was less soluble in general than GluA1. NLGN1 and 2 displayed similar solubility in four lysis conditions except in Doc (pH 8.0). Therefore, the protein solubility of NMDARs, AMPARs, and NLGNs expressed in HEK293T was affected by lysis conditions. In particular, the biochemical properties of NMDAR subunits in HEK293T cells, as in brain tissue, are sensitive to detergent, pH, and temperature.

### 3.7 The NMDAR Solubility Is Regulated by Its Cytosolic C‐Terminal Tail

**Figure 7:**
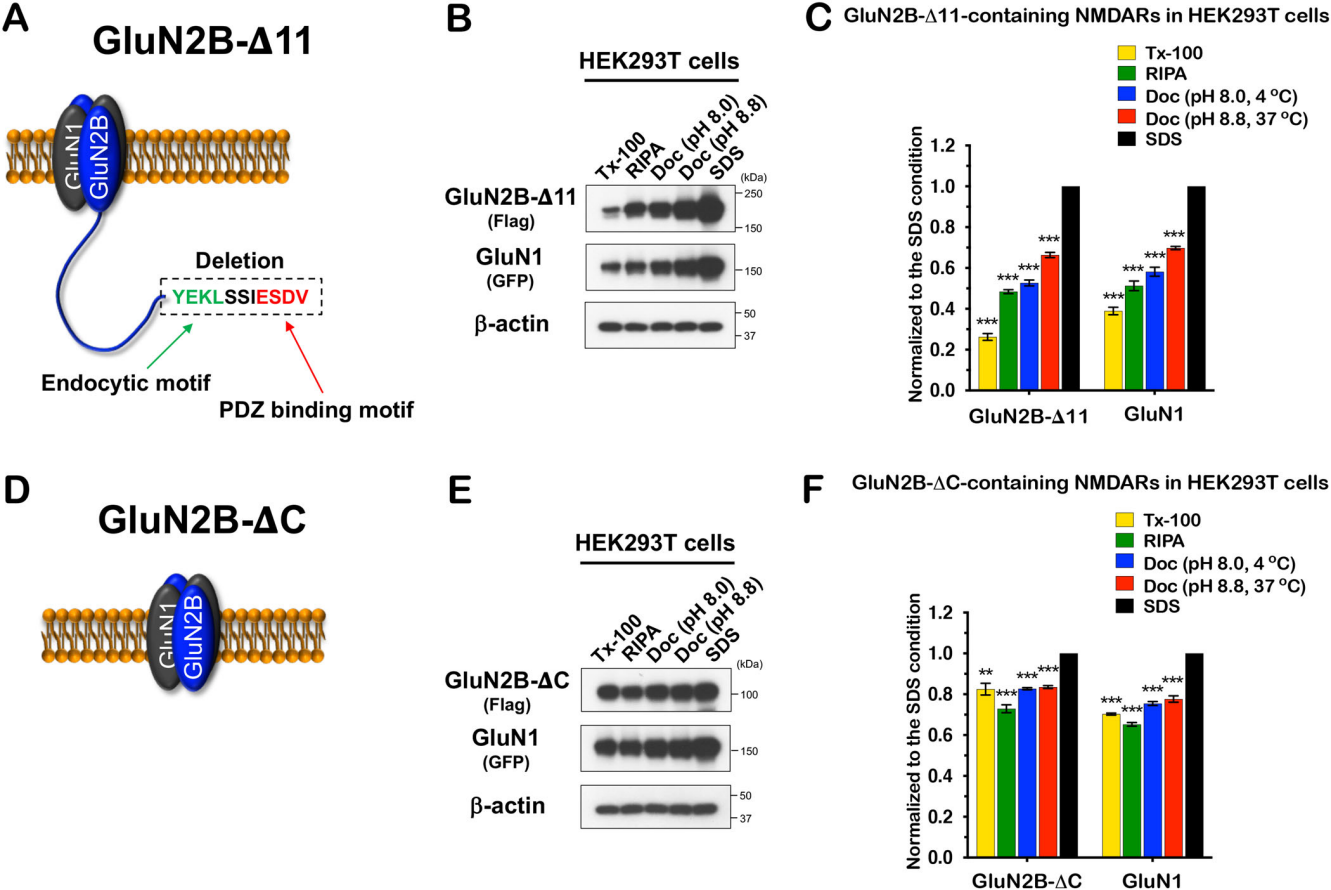
NMDAR solubility is regulated by the cytosolic tail. (A–F) GluN1 was cotransfected with GluN2B‐Δ11 or ‐ΔC in HEK293T cells. One day after transfection, protein samples were isolated in five different lysis conditions, then immunoblotted for NMDAR subunits using Flag or GFP antibody. Each condition was normalized to the SDS condition. (A) and (D) show cartoons depicting the GluN2B deletion mutant‐containing NMDARs. In (A), the last 11 amino acids containing the endocytic motif YEKL in green and the PDZ binding motif ESDV in red in cytosolic tail of GluN2B were deleted. In (C), Tx‐100 (*p* = 7.9E−07), RIPA (*p* = 3.9E−07), Doc (pH 8.0) (*p* = 2.7E−06), Doc (pH 8.8) (*p* = 6.0E−06) in GluN2B‐Δ11. Tx‐100 (*p* = 2.5E−06), RIPA (*p* = 1.6E−05), Doc (pH 8.0) (*p* = 2.3E−05), Doc (pH 8.8) (*p* = 1.7E−06) in GluN1. In (D), a stop codon was introduced into amino acid E_839_ of GluN2B and 644 amino acids of the cytosolic tail of GluN2B were deleted. In (F), Tx‐100 (*p* = 0.0018), RIPA (*p* = 7.3E−05), Doc (pH 8.0) (*p* = 2.6E−06), Doc (pH 8.8) (*p* = 9.3E−06) in GluN2B‐ΔC. Tx‐100 (*p* = 2.2E−07), RIPA (*p* = 1.2E−06), Doc (pH 8.0) (*p* = 7.7E−06), Doc (pH 8.8) (*p* = 6.8E−05) in GluN1. All experiments were repeated three times independently and the band intensities were quantified using ImageJ and analyzed statistically. All blots were normalized to the SDS condition. Error bars represent ± SEM, *p < 0.05, **p < 0.01, ***p < 0.001, *p* value is compared to the SDS condition.

NMDAR GluN2A and 2B subunits have large C‐tails of over 600 amino acids, whereas AMPAR subunits and NLGNs have short C‐tails of less than 100 amino acids. In addition, the C‐tails of NMDAR subunits include binding regions for many synaptic proteins (e.g., CaMKII, PSD‐95, SAP102, Fyn, and STEP_61_) as well as motifs for posttranslational modifications such as phosphorylation, palmitoylation, and ubiquitination. To investigate the role of the C‐tail in the solubility of NMDAR subunits in heterologous cells, we generated two truncation mutants of GluN2B: (1) GluN2B‐Δ11, which removes the YEKL endocytic motif and the PDZ ligand, in C‐terminal end; and (2) GluN2B‐ΔC, which removes the entire C‐tail by introducing a stop codon into amino acid E_839_ in GluN2B (Figure 7A,D). The endocytic and PDZ binding motifs of GluN2B are important for GluN2B‐containing NMDAR trafficking, localization, and surface expression [44]. Intriguingly, the solubility of GluN2B‐Δ11‐containing NMDAR in HEK293T cells using five different lysis buffers (Figure 7B,C) is not significantly changed when compared to GluN2B WT (Figure 6C,D). Surprisingly, the solubility of GluN2B and GluN1 subunits of GluN2B‐ΔC‐containing NMDAR in Tx‐100 lysis buffer is increased to 82.5% and 70.3%, respectively (Figure 7E,F). These data indicate that the C‐tail of GluN2B regulates GluN2B‐containing NMDAR solubility.

## 4 Discussion

Synapses are composed of numerous proteins that are required to develop and maintain synaptic structure and mediate synaptic transmission. Many proteins are concentrated and localized to the PSD including ion channels, glutamate receptors, scaffolding proteins, cell adhesion proteins, and actin‐binding proteins. The elucidation of the mechanisms regulating the synaptic targeting and precise localization of postsynaptic proteins has been central to improving our understanding of synaptic development, transmission, and plasticity. Postsynaptic proteins interact to form large multiprotein complexes [45, 46]. To study synaptic function, biochemical approaches including mass spectrometry have been applied to describe and understand PSD interactomes. In using mass spec, it is critical to take the biochemical properties of PSD proteins into consideration, most notably detergent solubility.

It has been recognized for many years that synaptic proteins have differing biochemical properties, including solubility. In fact, the PSD has traditionally been defined both morphologically by EM structure and biochemically by relative protein insolubility. To isolate the PSD proteins, biochemists use protocols of successive washes of increasing stringency. Therefore, when applying mass spec techniques, what conditions are the most useful? Those that keep most protein interactions intact, but likely leave many synaptic proteins behind in the insoluble fraction (e.g., Tx‐100) or harsher conditions that solubilize the majority of PSD proteins, but likely disrupt lower affinity protein‐protein interactions? Our study set out to define the solubility parameters of multiple types of synaptic proteins in varying conditions and found that there are profound differences that should be considered when designing a study.

Our findings revealed that synaptic proteins isolated from cultured neurons and differentiated cultured human forebrain neurons are typically more soluble than from rodent brain tissue. NLGNs are a good example of solubility doubling when isolated from cultured neurons or human forebrain neurons compared to tissue. Both NMDARs and PSD‐95 display increased solubility from cultured neurons and human forebrain neurons, whereas AMPARs are the same or even slightly decreased in cultured neurons compared to brain tissue. We provide Tables 1, 2, 3 to directly compare the different conditions and ideal solubilization of different proteins.

We also characterized the solubility of glutamate receptors expressed in heterologous cells because this is a common approach to evaluate receptor trafficking and function. We found that NMDARs are highly insoluble in HEK293T cells, which may seem counterintuitive. Traditionally, biochemical preparations from brain tissue equate Tx‐100 insolubility with the PSD and with this logic, expression in HEK293T cells should not have a significant insoluble fraction. Therefore, it is important to note that the relative insolubility of NMDARs is apparent in heterologous systems lacking neuronal synapses. Indeed, even AMPARs and NLGNs, which are considered highly soluble in Tx‐100 display differences in this preparation. One obvious structural difference between NMDARs, AMPARs, and NLGNs is that the NMDAR subunits have a long C‐tail, whereas AMPARs and NLGNs have short C‐tails. Interestingly, truncation of the entire C‐tail of GluN2B increased the solubility of NMDARs expressed in HEK293T cells, whereas an 11 amino acid deletion of GluN2B, which removes the PDZ binding motif and endocytic motif, did not increase its solubility. GluN2B C‐tail also has many posttranslational modification sites such as phosphorylation and palmitoylation. In particular, palmitoylation is highly associated with protein localization to specific subcellular regions such as synaptic membranes or lipid rafts. In addition, it is known that palmitoylation regulates NMDAR trafficking [47, 48, 49]. These data suggest that the entire C‐tail truncation of GluN2B might be attributable to changes in posttranslational modifications. Understanding the mechanisms underlying the C‐tail effect on solubility and potential concomitant effect on trafficking will be a topic of future studies.

Newer approaches such as differentiated iPSCs are often considered a more accurate model of in vivo endogenous expression. However, protocols vary widely. Many different induction methods are used to differentiate neurons from iPSCs or develop organoids. These preparations are gaining popularity in the study of molecular mechanisms underlying or physiological conditions associated with human diseases such as NDDs. In this study, we used a multistep differentiation protocol that includes a dual‐smad inhibition method to obtain forebrain neurons from iPSCs. For the first time, we investigated the solubility of a variety of synaptic proteins in human forebrain neurons from iPSCs using five different protein preparation methods. Interestingly, NMDAR subunits in human forebrain neurons are not soluble in Tx‐100 lysis buffer when compared to AMPARs, NLGNs, or PSD‐95 but NMDAR subunits in Tx‐100 are more soluble in human forebrain neurons than in rat primary cultured neurons. These data show that proteins in human neurons displayed increased solubility compared to hippocampal tissue and rat‐cultured neurons. The observation that synaptic proteins, particularly NMDARs and PSD‐95, in human neurons are more soluble than in hippocampal tissues and rat‐cultured neurons is striking and the reason is unclear. Indeed, very little is known about the biology of human‐differentiated neurons. They have not been studied using comprehensive biochemical approaches to identify sophisticated synaptic protein complexes nor has synaptic structure been studied carefully. In fact, the most popular protocols using single gene induction result in human‐differentiated neurons that fail to express glutamate receptors. Our findings will provide the fundamental characterization needed to investigate the synaptic protein complexes and glutamate receptor trafficking in human neurons.

Overall, we have conducted an extensive biochemical characterization of synaptic proteins. Because there are hundreds of proteins at synapses, we opted to take a few representative postsynaptic proteins including receptors, adhesion molecules, and scaffolding proteins. As with previous studies [35, 50, 51], we showed that NMDARs are more insoluble than AMPARs. However, we extended these analyses to include different cell preparations and observed some unexpected results. We hope these findings will help guide future studies on postsynaptic protein complexes and interactomes.

## Author Contributions

Sehoon Won and Katherine W. Roche designed research. Sehoon Won and Colin L. Sweeney performed experiments. Sehoon Won and Katherine W. Roche analyzed data. Sehoon Won, Colin L. Sweeney, and Katherine W. Roche wrote the paper.

## Conflicts of Interest

The authors declare no conflicts of interest.

## Supporting information

Figure S1. Temperature of protein preparation affects NMDAR detection on immunoblot. Synaptic proteins in cultured cortical neurons were isolated at *DIV* 28 using 1% SDS lysis buffer and same amount of protein was prepared at two different temperatures 42°C and 95°C, then immunoblotted with indicated antibodies.

## Data Availability

The data that support the findings of this study are available from the corresponding author upon reasonable request.
