# Supplementary figures and images for: Biochemical Properties of Synaptic Proteins Are Dependent on Tissue Preparation: NMDA Receptor Solubility Is Regulated by the C‐Terminal Tail
Source: J Cell Biochem. 2024 Oct 6;126(1):e30664. doi: 10.1002/jcb.30664 (PMC11730348; doi:10.1002/jcb.30664)

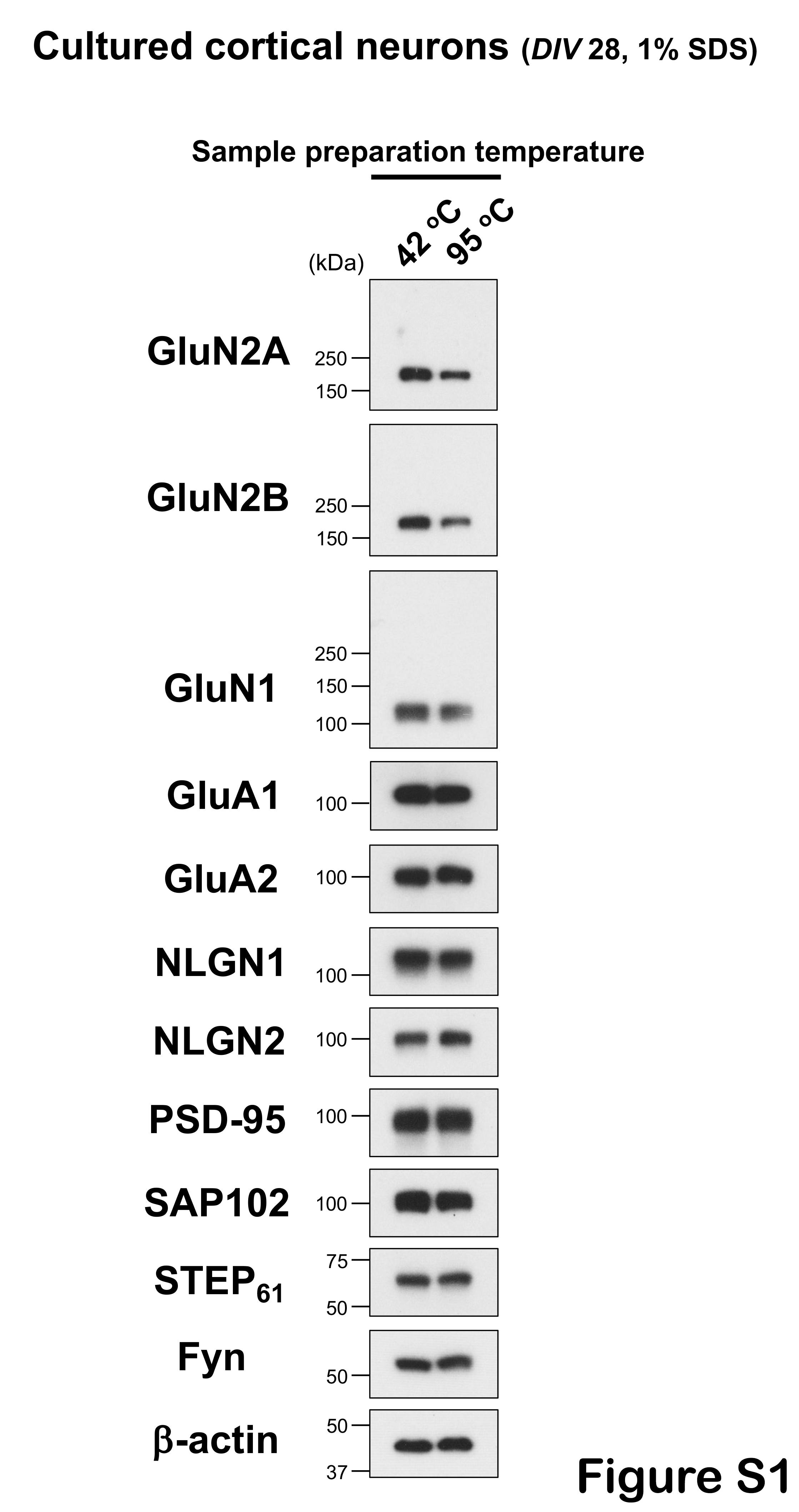

Supplement: Supplementary file 1 — Figure S1. Temperature of protein preparation affects NMDAR detection on immunoblot. Synaptic proteins in cultured cortical neurons were isolated at DIV 28 using 1% SDS lysis buffer and same amount of protein was prepared at two different temperatures 42°C and 95°C, then immunoblotted with indicated antibodies. [file JCB-126-e30664-s001.tif]
